# The short-term cost-effectiveness of once-weekly semaglutide versus once-weekly dulaglutide for the treatment of type 2 diabetes mellitus in Colombian adults

**DOI:** 10.12688/f1000research.128441.1

**Published:** 2023-07-31

**Authors:** Hans Liebisch-Rey, Andrea-Marcela Suarez-Chacon, Yuli-V. Fuentes, Jhosep Blanco, Joshua Kock, Sharon Lechtig-Wassermann, Rosa Helena Bustos

**Affiliations:** 1 Evidence-Based Therapeutics Group, Department of Clinical Pharmacology, Faculty of Medicine, Universidad de La Sabana and Clinica Universidad de La Sabana, Chía, Cundinamarca, 140013, Colombia; 2 Department of Epidemiology, Faculty of Medicine, Universidad de La Sabana, Chía, Cundinamarca, 140013, Colombia

**Keywords:** Semaglutide, Dulaglutide, Pharmacoeconomic, Cost-effectiveness

## Abstract

**Background**: Type 2 Diabetes Mellitus (T2DM) is a highly prevalent disease worldwide and in Colombia, representing one of the main causes of death and placing a considerable burden on healthcare systems. 13 classes of drugs are approved for the treatment of T2DM, with Glucagon-like Peptide-1 (GLP-1) receptor agonists being a first-line treatment option for patients with or at high risk of certain cardiovascular diseases and chronic kidney disease. The objective of this study is to conduct a short-term cost-effectiveness analysis of once-weekly semaglutide versus once-weekly dulaglutide in Colombian adults with T2DM, from a third-party payer perspective. **Methods:** Numbers needed to treat were calculated for different single and composite endpoints of the SUSTAIN 7 trial, annual costs for once weekly semaglutide 1.0 mg and dulaglutide 1.5 mg were extracted from the public SISMED database. With these inputs a cost of control model was developed, to obtain the annual cost of bringing one T2DM patient to relevant clinical outcomes by using semaglutide or dulaglutide. **Results:** Semaglutide was considered cost-effective compared to dulaglutide across all pre-specified endpoints, even in the different scenarios evaluated in the sensitivity analyses, and in a particularly pronounced manner for weight loss outcomes. Semaglutide at a dose of 1.0 mg once-weekly was cost-effective compared to dulaglutide 1.5 mg across all outcomes in the short-term, making it an appropriate first-line choice in the treatment of T2DM when deciding between these two GLP-1 receptor agonists. **Conclusions:** This is the first short-term cost-effectiveness study of semaglutide and dulaglutide in T2DM Colombian patients. Our modeled results suggest that once-weekly semaglutide represents a cost-effective option for treating individuals with T2DM in Colombia who are not achieving glycaemia control with metformin, and it would be expected to improve HbA1C, promote greater weight loss and reduce costs from a third-payer perspective compared with treatment with dulaglutide.

## Introduction

An estimated 537 million people were living with diabetes worldwide in 2021, and this number is expected to increase based on trends and future projections, suggesting that by 2045 the absolute number of people with diabetes will have increased by 46%. The global prevalence of diabetes is estimated to be over 10%, with the highest prevalence rate observed in low and middle-income countries, meaning that three out of four adults with diabetes live in these regions. ^1^ In Colombia, the prevalence for type 2 diabetes mellitus (T2DM) ranges from 7.1%-8.5% overall, with wide variations between rural areas (1.4%-7.9%) and urban locations (1%-46%), representing the fifth leading cause of death with a rate of 15 deaths per 100,000 individuals. ^2^ This is a worrying finding, as the burden of diabetes is accompanied by large healthcare expenditures, accounting for 966 billion USD worldwide and 2.6 billion dollars annually in Colombia. ^1^^,^^2^

Until recently, 12 classes of drugs were approved to treat T2DM, with a further option -the dual targeted tirzepatide-, receiving Food and Drug and Administration (FDA) approval in May 2022. ^3^^,^^4^ These treatments are either oral or injectable, aiming to prevent or delay the occurrence of microvascular and macrovascular complications, the main causes of morbidity and mortality in patients with diabetes. ^4^ Enhanced glycemic control can be achieved with glucagon-like peptide-1 (GLP-1) receptor agonists, recommended by the American Diabetes Association (ADA) and the European Association for the Study of Diabetes (EASD) as a first-line treatment option for individuals with T2DM with or at high risk for cardiovascular disease, heart failure, and/or chronic kidney disease. Furthermore, in these patients a GLP-1 receptor agonist is recommended over insulin when possible, and it is also the preferred addition to basal insulin for combined injection therapy. ^5^

Multiple studies have shown robust evidence with this drug class for cardiovascular benefits among patients with T2DM. A systematic review with meta-analysis that included seven clinical trials showed that, overall, the GLP-1 receptor agonist family reduced major adverse cardiovascular events (MACE), including cardiovascular death, stroke, or myocardial infarction, by 12%. ^6^ Similar findings were also shown in another meta-analysis, demonstrating that GLP-1 receptor agonist treatment showed a significant 10% relative risk reduction in the three-point major adverse cardiovascular event primary outcome (cardiovascular mortality, non-fatal myocardial infarction, and non-fatal stroke), and a 12% relative risk reduction in all-cause mortality. ^7^ Once-weekly semaglutide and dulaglutide are GLP-1 receptor agonists approved for the treatment of T2DM by the FDA, European Medicines Agency (EMA) and the Colombia National Food and Drug Surveillance Institute (INVIMA), ^8^^,^^9^ with demonstrated efficacy in the SUSTAIN clinical trial program for the former and the AWARD trial program for the latter. ^10^^,^^11^

The purpose of our study was to conduct a short-term cost-effectiveness analysis of once-weekly semaglutide versus once-weekly dulaglutide in Colombian adults with T2DM, from a third-party payer perspective, as has been recommended in multiple methodological guidelines for economic evaluations. ^12^

## Methods

### Ethical compliance

This article is based on previously conducted studies and does not contain any studies with human participants or animals performed by any of the authors. It is considered research without ethical risks, in accordance with resolution 8430 of 1993 of the Colombian Ministry of Health. ^13^

**Figure 1.**
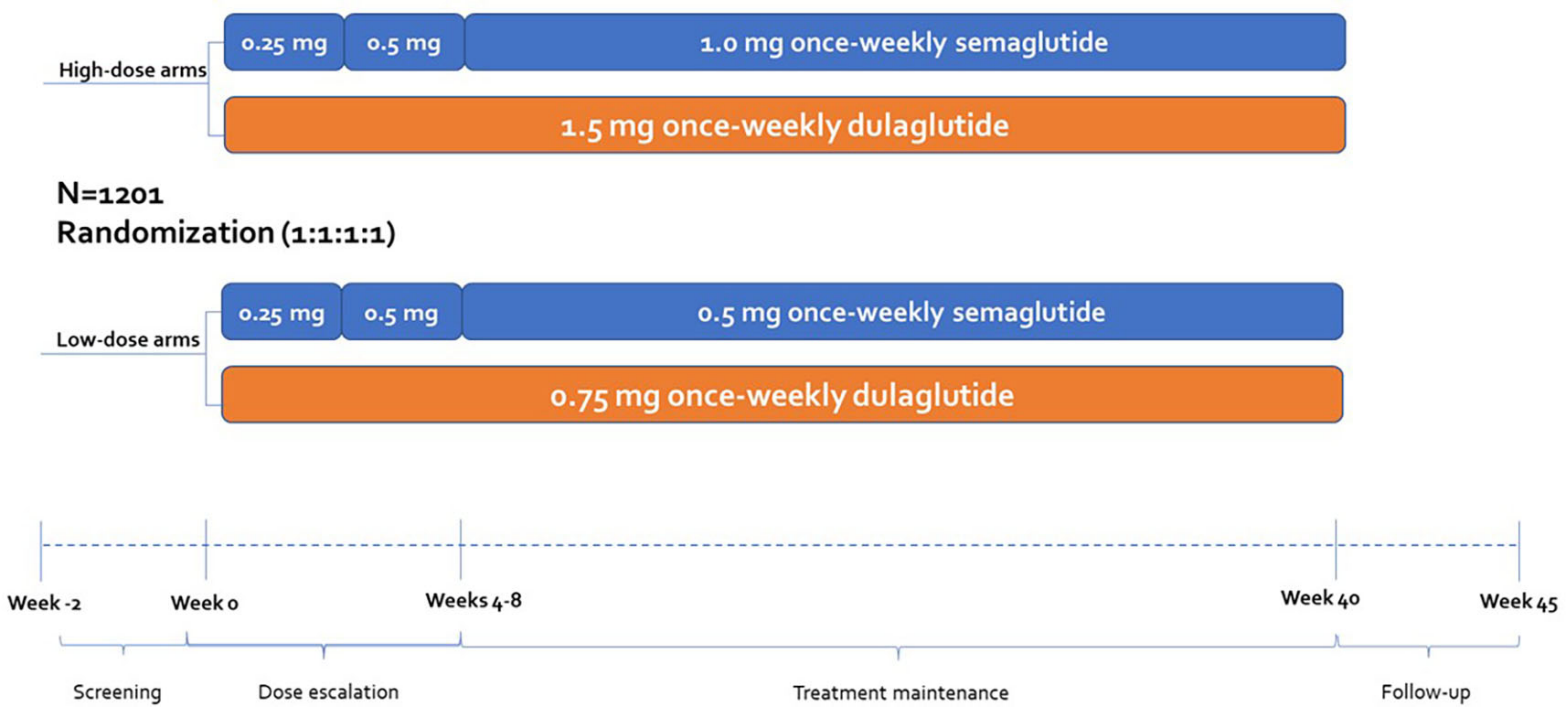
Design of the SUSTAIN 7 randomized controlled trial.

A cost of control model was created using Microsoft Excel to assess To assess numbers needed to treat (NNT) as well as relative and absolute costs according to the criteria of SUSTAIN 7, randomized controlled trial ( Figure 1 and Table 1). SUSTAIN 7 considers clinical parameters such as weight, and blood glucose and hypoglycemia results ( Table 1).

**Table 1.** Proportion of patients reaching target with once weekly semaglutide 1.0 mg, and dulaglutide 1.5 mg, all in combination with metformin, in the SUSTAIN 7 trial.

Endpoint	Once-weekly semaglutide 1.0 mg (%)	Once-weekly dulaglutide 1.5 mg (%)
HbA1c <7.0%	79	67
HbA1c ≤6.5%	67	47
HbA1c <7.0% without hypoglycemia, and no weight gain	74	58
Weight loss ≥5%	63	30
Weight loss ≥10%	27	8
≥1.0% HbA1c reduction and ≥3.0% weight loss	68	35

## Results

The higher doses contained in SUSTAIN 7 such as semaglutide 1 mg and dilaglutide 1.5 were used for modeling in this study ( Table 2) ^14^ with n=600 patients.

**Table 2.** Example cost of control calculation based on the proportion of patients achieving a HbA1c target <7%.

	Semaglutide 1.0 mg/once-weekly	Dulaglutide 1.5 mg/once-weekly	Interpretation
Drug cost (COP)/year	$ 5.843.354,89	$ 5.883.774,07	
Drug cost index	0,993130399	1,006917119	Price maintenance between semaglutide 1.0 mg versus dulaglutide 1.5 mg once weekly
NNT to achieve HbA1C <7.0%	1,27	1,49	
Cost per patient achieving control (COP)	$ 7.421.060,72	$ 8.766.823,37	
Amount spent (according to achieve target relative/once-weekly semaglutide)	1	1,181343706	The proportion of money spent is 1.18 times (18%) higher for delaglutide 1.5 mg compared to semaglutide 1.0. (Target patient HbA1C <7.0%). That is, for each $100.000 COP spent on semaglutide, $118.000 COP would be spent on dulaglutide to achieve this outcome.

The perspective of a third payer in Colombia was assumed over a timeline of one year for this study for two diabetes medication third payer in Colombia for one year. For this reason, discount values were not used either. 40 weeks of follow-up data from SUSTAIN 7 were taken into account to determine efficacy and were not extrapolated beyond the trial period. This allowed the reduction of the uncertainty of the modeled results. Drug prices for once weekly semaglutide 1.0 mg, and dulaglutide 1.5 mg were based on the 2021 costs derived from the SISMED database (Medication Price Information System, by its acronym in Spanish), which includes information on the prices of essential medicines in Colombia. ^15^ No other cost data was taken into account for the analysis in our study and 100% adherence was assumed for the two drugs once a week.

The NNT was calculated in absolute terms for each comparator. For placebos, NNTs are calculated assuming that zero patients in the group being compared achieve the specified outcome ( Table 3). The absolute cost of control was calculated by multiplying the annual cost of treatment for each medication by the NNT of the selected data. The conservative approach was from the once-weekly semaglutide perspective, addressing a full year of treatment costs, thus extending beyond 40 weeks of SUSTAIN 7. Relative costs of control were calculated by reference to the cost of control at semaglutide 1.0 mg once a week.

**Table 3.** NNT calculation for one of the SUSTAIN 7 outcomes. ARR=Absolute Risk Reduction.

Example for HbA1C <7.0%	NNT calculation
Semaglutide 1.0 mg *	ARR=79% - 0%=79%|NNT=1/ARR=1/0.79=1.27
Dulaglutide 1.5 mg *	ARR=67% - 0%=67%|NNT=1/ARR=1/0.67=1.5

* Once-weekly.

One-way sensitive analyses were performed around the base case, such as varying the percentage of patients meeting each target by an approximation of the standard error (SE). This was done with the following formula in equation 1, where *n* is the number of patients in the arm of SUSTAIN 7 and *p* is the percentage of patients achieving each endpoint:

$$\sqrt{\frac{1}{n}p\left(1-p\right)}$$ (1)

Additionally, cost of control calculations were performed for the best- and worst-case pricing scenarios for semaglutide –the best scenario being when costs are the lowest possible for semaglutide and the highest for dulaglutide, and the worst being the opposite situation-, taking into account the range of prices (minimum and maximum) that were obtained from the SISMED database.

## Results

### Base case annual costs

The annual base cost for semaglutide and dulaglutide were calculated using the SISMED database, with similar costs per patient for both medications. Semaglutide 1.0 mg had a monthly cost of $486.946 Colombian pesos ($5.843.355 annually), while dulaglutide 1.5 mg had a monthly cost of $489.762 Colombian pesos ($5.877.144 pesos annually) ( Table 4). ^22^

**Table 4.** Colombian drug prices per month of treatment according to SISMED in 2021 Colombian pesos (COP) (December 2021).

Glucagon-like peptide 1 treatment	Pack contents (mg)	Number of pens	Base pack price (COP)	Minimum pack price (COP)	Maximum pack price (COP)
Semaglutide 1.0 mg	4	1	$ 486.946,24	$ 479.403,52	$ 503.170,95
Dulaglutide 1.5 mg	6	4	$ 489.762,03	$ 484.818,30	$ 490.314,51

### Numbers needed to treat (NNT)

**Figure 2.**
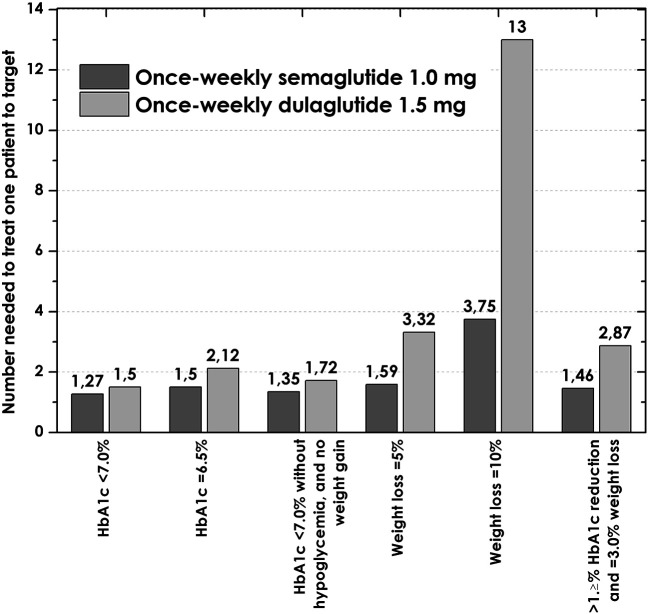
Numbers needed to treat to bring one patient to target with once-weekly semaglutide 1.0 mg, and dulaglutide 1.5 mg. HbA1C, glycated hemoglobin.

The NNTs for all outcomes were larger for dulaglutide, with the most important differences compared to semaglutide being in the weight loss outcomes, particularly for achieving a weight loss ≥10%, with an NNT of 3.7 for semaglutide and 12.5 for dulaglutide ( Figure 2). ^22^

### Cost of control

**Figure 3.**
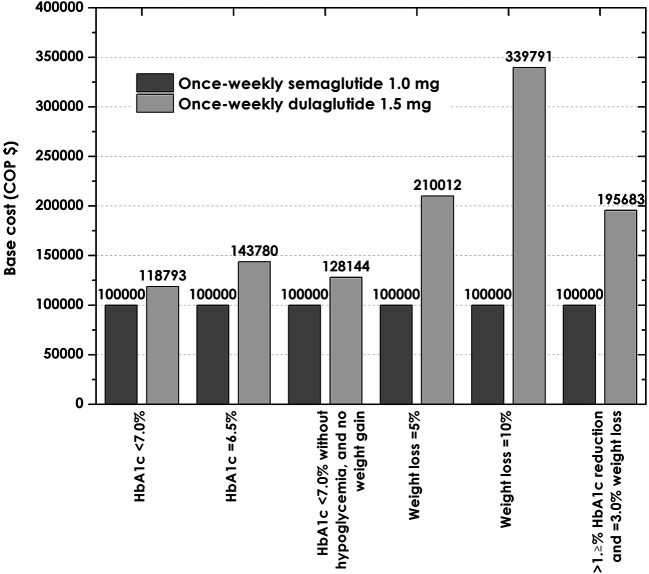
Relative cost once weekly for semaglutide 1.0 mg and dulaglutide 1.5 mg compared to base case (index=100,000) once weekly. HbA1C, glycosylated hemoglobin.

The previous values were used to estimate the cost per patient successfully reaching the SUSTAIN 7 outcomes. Six SUSTAIN 7 endpoints allowed calculation of the absolute cost of control for the high-dose arms of the drug. According to Table 5, the cost to achieve control was less for the drug semaglutide. Regarding the composite outcome of HbA1c <7%, no weight gain, and no hypoglycemia, which is particularly important -this HbA1C goal is considered appropriate for most T2DM patients by the ADA 2022 guidelines, ^16^ when it is not associated with significant hypoglycemia-, the cost per patient reaching this endpoint was $7.888.529 for semaglutide, compared to $10.108.687 with dulaglutide. This means that an expenditure 28% greater would have to be spent on dulaglutide to bring one patient to this target ( Figure 3). ^22^

**Table 5.** Absolute annual cost of control outcomes with once-weekly semaglutide 1.0 mg, and dulaglutide 1.5 mg in 2021 Colombian pesos (COP). Calculations in the base cost, best and worst scenarios for semaglutide.

Endpoint	Base cost		Best scenario for semaglutide		Worst scenario for semaglutide	
	Once-weekly semaglutide 1.0 mg (COP)	Dulaglutide 1.5 mg (COP)	Once-weekly semaglutide 1.0 mg (COP)	Dulaglutide 1.5 mg (COP)	Once-weekly semaglutide 1.0 mg (COP)	Dulaglutide 1.5 mg (COP)
HbA1c <7.0%	$ 7.421.060,85	$ 8.756.944,56	$ 7.306.109,67	$ 8.766.823,37	$ 7.668.325,21	$ 8.668.551,16
HbA1c ≤6.5%	$ 8.706.598,95	$ 12.518.316,72	$ 8.571.734,97	$ 12.532.438,78	$ 8.996.696,50	$ 12.391.955,68
HbA1c <7.0% without hypoglycemia, and no weight gain	$ 7.888.529,25	$ 10.108.687,68	$ 7.766.337,05	$ 10.120.091,41	$ 8.151.369,32	$ 10.006.649,66
Weight loss ≥5%	$ 9.290.934,45	$ 19.570.889,52	$ 9.147.019,20	$ 19.592.967,67	$ 9.600.501,64	$ 19.373.339,17
Weight loss ≥10%	$ 21.620.413,50	$ 73.464.300,00	$ 21.285.516,37	$ 73.547.175,94	$ 22.340.789,98	$ 72.722.744,62
≥1.0% HbA1c reduction and ≥3.0% weight loss	$ 8.589.731,85	$ 16.808.631,84	$ 8.456.678,13	$ 16.827.593,85	$ 8.875.935,48	$ 16.638.963,97

### Sensitivity analyses

**Figure 4.**
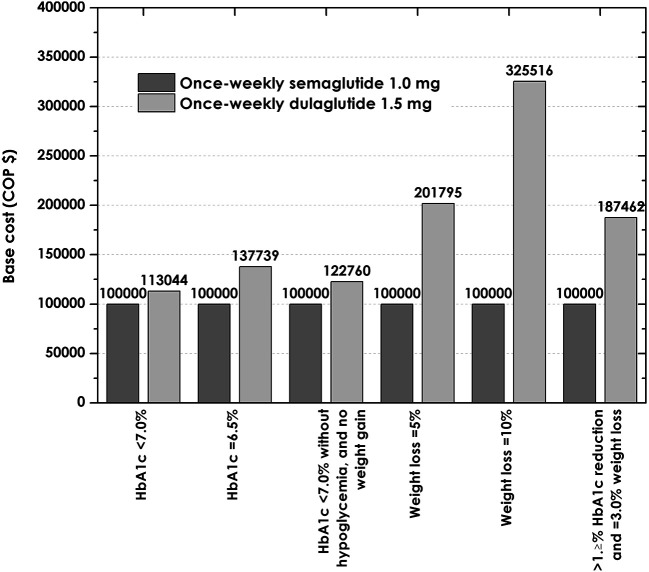
Relative cost of control with once-weekly semaglutide 1.0 mg and dulaglutide 1.5 mg versus once-weekly in the worst scenario of cost for semaglutide (index = 100.000). HbA1C, glycated hemoglobin.

Sensitivity analyses showed that variations in cost assumptions, where worst case scenarios for semaglutide (those where the price of this drug was highest and that of dulaglutide was lowest) were considered, did not change the finding that the cost of reaching the composite endpoint with semaglutide was lower than the cost with dulaglutide after one year of treatment, with semaglutide having a cost per patient of $8.151.369, compared to $10.006.649 with dulaglutide, representing an expenditure 22% higher ( Figure 4). Considering the one-way sensitivity, we reduced that by decreasing the proportion of patients reaching targets by one standard error (SE) with semaglutide once a week and increasing the patients reaching this target with dulaglutica by one SE, the cost of control of semaglutide once a week was lower by endpoints ( Table 5). In this way, these analyzes support the conclusions of the base case analysis.

## Discussions

We conducted an evaluation of the short-term cost-effectiveness of the GLP-1 receptor agonists semaglutide and dulaglutide, with cost-effectiveness assessed through the development of a cost of control model, in order to evaluate the numbers needed to treat (NNT) as well as the absolute and relative costs of bringing a single patient to each of the pre-specified composite and single endpoints in the SUSTAIN 7 trial, which demonstrated a higher efficacy with semaglutide for all outcomes. ^14^

The calculations from our analysis suggest that achieving clinically relevant endpoints from SUSTAIN 7 would result in economic savings with once weekly semaglutide, compared to dulaglutide after one year of treatment. The annual drug cost for both medications was similar in the base case, with our results being consistent with previous studies that have also demonstrated that semaglutide is a cost-effective option when compared to dulaglutide. ^17^^–^^21^ In this study, we synthesized the effectiveness and expenditure evidence and found that semaglutide was associated with the lowest cost per patient reaching disease control for all endpoints, findings that were reaffirmed in our sensitivity analyses.

This study has important implications for stakeholders considering this is the first cost-effectiveness analysis to date comparing subcutaneous semaglutide and dulaglutide in the Colombian diabetic population, potentially allowing a better allocation of resources. Previous studies have demonstrated the superiority of semaglutide over dulaglutide in both short and long-term cost-effectiveness analyses, most of them being carried out in high-income countries. ^17^^–^^21^

This study has several limitations. First, we restricted our comparison to semaglutide *versus* dulaglutide. It is important to acknowledge that there are other available molecules in the Colombian market. Second, we limited the costs in the analysis to drugs, as they were expected to be the major drivers of the cost-effectiveness of semaglutide and dulaglutide. Therefore, this model did not account for all potential costs. Third, the analysis takes a third-payer perspective over a short-term horizon. Alternative perspectives and time horizons may result in variable cost-effectiveness estimations, and as such, additional cost-effectiveness studies for these molecules with longer time horizons would be a welcome complement for our study. Fourth, we included prices disregarding potential discounts or refunds, which payers might need to consider in their decision-making processes.

## Conclusions

This is the first short-term cost-effectiveness study of semaglutide and dulaglutide in T2DM Colombian patients. Our modeled results suggest that once-weekly semaglutide represents a cost-effective option for treating individuals with T2DM in Colombia who are not achieving glycaemia control with metformin, and it would be expected to improve HbA1C, promote greater weight loss and reduce costs from a third-payer perspective compared with treatment with dulaglutide. Additional cost-effectiveness studies are war-ranted to evaluate the long-term cost-effectiveness of these molecules in the Colombian diabetic population.

## Data Availability

All data underlying the results are available as part of the article and no additional source data are required. Zenodo: The short-term cost-effectiveness of once-weekly semaglutide versus once-weekly dulaglutide for the treatment of type 2 diabetes mellitus in Colombian adults. https://doi.org/10.5281/zenodo.7857437. ^22^ This project contains the following underlying data:
• Final Data_F1000.xlsx. (Semaglutide and dulaglutide cost calculations for this study). Data are available under the terms of the Creative Commons Attribution 4.0 International license (CC-BY 4.0).
