# Supplementary material for: The short-term cost-effectiveness of once-weekly semaglutide versus once-weekly dulaglutide for the treatment of type 2 diabetes mellitus in Colombian adults
Source: F1000Res. 2023 Dec 11;12:914. Originally published 2023 Jul 31. [Version 2] doi: 10.12688/f1000research.128441.2 (PMC10730985; doi:10.12688/f1000research.128441.2)
Supplement: Peer review history

# Reviewer report: Reviewer response for version 2

**DOI:** 10.5256/f1000research.159773.r229096

**Published:** 2023-12-19

**Authors:** George Gourzoulidis

**Affiliations:** 1 Health Through Evidence, Athens, Greece

no other comments

Is the work clearly and accurately presented and does it cite the current literature?

Yes

If applicable, is the statistical analysis and its interpretation appropriate?

Partly

Are all the source data underlying the results available to ensure full reproducibility?

Yes

Is the study design appropriate and is the work technically sound?

Partly

Are the conclusions drawn adequately supported by the results?

Yes

Are sufficient details of methods and analysis provided to allow replication by others?

No

Reviewer Expertise:

Economic EvaluationHealth Services ResearchOutcomes ResearchHealth Technology AssessmentHealthcare Economics

I confirm that I have read this submission and believe that I have an appropriate level of expertise to confirm that it is of an acceptable scientific standard.

# Reviewer report: Reviewer response for version 1

**DOI:** 10.5256/f1000research.141032.r218541

**Published:** 2023-11-22

**Authors:** Sajad Hamal, Ahmed Ghanem

**Affiliations:** 1 The Lundquist Institute, Torrance, California, USA; 2 Cardiology, The Lundquist Institute, Torrance, California, USA

An interesting analysis, however, the generalizability may be limited, as the prices and cost basis for each therapy is based on prices in Columbia. Clearly, Semaglutide is more potent at the doses chosen, so will demonstrate dominance for each outcome, based on lower NNT and similar price structures. However, both Dulaglutide and Semaglutide come in higher doses now, so that is worthy of discussion and consideration of secondary analysis. At least discussing the more potent versions of each drug now available worldwide.

Is the work clearly and accurately presented and does it cite the current literature?

Yes

If applicable, is the statistical analysis and its interpretation appropriate?

Yes

Are all the source data underlying the results available to ensure full reproducibility?

Yes

Is the study design appropriate and is the work technically sound?

Partly

Are the conclusions drawn adequately supported by the results?

Yes

Are sufficient details of methods and analysis provided to allow replication by others?

Yes

Reviewer Expertise:

Cardiology

We confirm that we have read this submission and believe that we have an appropriate level of expertise to confirm that it is of an acceptable scientific standard.

# Reviewer report: Reviewer response for version 1

**DOI:** 10.5256/f1000research.141032.r204359

**Published:** 2023-09-18

**Authors:** George Gourzoulidis

**Affiliations:** 1 Health Through Evidence, Athens, Greece

1. "An estimated 537 million people were living with diabetes worldwide in 2021, and this number is expected to increase based on trends and future projections, suggesting that by 2045 the absolute number of people with diabetes will have increased by 46%". Please provide refs.
2. Why is a cost-effectiveness analysis of once-weekly semaglutide versus once-weekly dulaglutide in Colombian adults with T2DM necessary?
3. Cost of control model - figure of the model? Can the authors describe the model?
4. The study is weak in methods sections, the authors have to present the cost inputs of the model. Moreover, since this is a cost-effectiveness, the authors have to report why they do not take into account the QALYS?
5. Why the one-year horizon was selected?
6. The results have to be separated to clinical and cost outcomes.

Is the work clearly and accurately presented and does it cite the current literature?

Yes

If applicable, is the statistical analysis and its interpretation appropriate?

Partly

Are all the source data underlying the results available to ensure full reproducibility?

Yes

Is the study design appropriate and is the work technically sound?

Partly

Are the conclusions drawn adequately supported by the results?

Yes

Are sufficient details of methods and analysis provided to allow replication by others?

No

Reviewer Expertise:

Economic EvaluationHealth Services ResearchOutcomes ResearchHealth Technology AssessmentHealthcare Economics

I confirm that I have read this submission and believe that I have an appropriate level of expertise to confirm that it is of an acceptable scientific standard, however I have significant reservations, as outlined above.
